# Item response theory analysis of cognitive tests in people with dementia: a systematic review

**DOI:** 10.1186/1471-244X-14-47

**Published:** 2014-02-19

**Authors:** Sarah McGrory, Jason M Doherty, Elizabeth J Austin, John M Starr, Susan D Shenkin

**Affiliations:** 1 Alzheimer Scotland Dementia Research Centre, University of Edinburgh, 7 George Square, Edinburgh EH8 9JZ, UK; 2 Psychology, University of Edinburgh, Edinburgh, UK; 3 Geriatric Medicine, University of Edinburgh, Edinburgh, UK; 4 Centre for Cognitive Ageing and Cognitive Epidemiology, University of Edinburgh, Edinburgh, UK

**Keywords:** Item response theory, Dementia, Psychometrics, Cognition, Alzheimer disease, MMSE, Systematic review

## Abstract

**Background:**

Performance on psychometric tests is key to diagnosis and monitoring treatment of dementia. Results are often reported as a total score, but there is additional information in individual items of tests which vary in their *difficulty* and *discriminatory* value. Item *difficulty* refers to an ability level at which the probability of responding correctly is 50%. *Discrimination* is an index of how well an item can differentiate between patients of varying levels of severity. Item response theory (IRT) analysis can use this information to examine and refine measures of cognitive functioning. This systematic review aimed to identify all published literature which had applied IRT to instruments assessing global cognitive function in people with dementia.

**Methods:**

A systematic review was carried out across Medline, Embase, PsychInfo and CINHAL articles. Search terms relating to IRT and dementia were combined to find all IRT analyses of global functioning scales of dementia.

**Results:**

Of 384 articles identified four studies met inclusion criteria including a total of 2,920 people with dementia from six centers in two countries. These studies used three cognitive tests (MMSE, ADAS-Cog, BIMCT) and three IRT methods (Item Characteristic Curve analysis, Samejima’s graded response model, the 2-Parameter Model). Memory items were most *difficult*. Naming the date in the MMSE and memory items, specifically word recall, of the ADAS-cog were most *discriminatory*.

**Conclusions:**

Four published studies were identified which used IRT on global cognitive tests in people with dementia. This technique increased the interpretative power of the cognitive scales, and could be used to provide clinicians with key items from a larger test battery which would have high predictive value. There is need for further studies using IRT in a wider range of tests involving people with dementia of different etiology and severity.

## Background

Global cognitive functioning measures are the mainstay diagnostic tool for dementia, in conjunction with determination of functional decline, and are also used to track and measure disease course. Measures of cognition in dementia should be able to both reliably detect the disease in its early stages and to evaluate the severity of the disease [1].

The most common method of scoring a cognitive test is to sum the raw score. The total score is used to aid diagnosis and to assess and monitor disease severity. This method is quick and simple to apply and is based on the premise of all test items reflecting a common unobservable trait or ability range along which cognitive impairment can be measured [2].

However the simple summation of raw scores overlooks any differences between the items and information the pattern of response can provide. It may therefore lead to an inaccurate estimation of cognitive impairment [2].

Items within a measure will differ in several ways. Firstly some items may be more *difficult* than others, for example, for most people, repeating a noun would be less *difficult* than remembering a phrase or list of words. Secondly, some items may be more sensitive to the early stages of cognitive decline and others to the later stages of the disease. Thirdly, items may differ in how sensitive they are to clinical change. Finally, some items may be redundant and provide no meaningful variability to the measure. These items could be removed to ease the burden on patients and clinicians.

The same total score can be achieved via many different patterns of response. For example, two individuals scoring 20 on the MMSE may have correctly and incorrectly answered completely different items. Likewise an individual obtaining the same total score before and after treatment would be considered as having experienced no change in cognitive impairment even if the pattern of response across the items had changed.

Therefore, there is a need to look beyond the total score and to investigate the pattern of response to the individual items. This can be done using the statistical method ‘item response theory’ (IRT) [3].

IRT is based on the probability of a person achieving a certain score on a test being a consequence of that person’s ability on the latent construct [4]. As that ability, cognitive function in this case, changes, so too does the probability of the individual achieving a certain score, offering measurement precision that varies with ability level [5]. Unlike other statistical methods which use the aggregate raw score as an indication of ability, IRT is more concerned with individual test items.

IRT can provide two useful measures; *difficulty* and *discrimination*, both of which are technical properties of the Item Characteristic Curve (ICC). The ICC is a non-linear regression on ability of probability of a correct response to each item. *Difficulty* is the ability value that is associated with a 50% probability of scoring one (rather than zero) on an individual item [6].

*Discrimination,* reflecting the slope of the ICC in its middle section, is an index of how well an item can differentiate between patients of varying levels of severity. More *discriminating* items, with a steeper slope, are better able to differentiate among individuals in the range of the latent trait [7].

The performance of the overall scale can be measured using the Test Characteristic Curve (TCC). The TCC is a valuable tool for assessing the range of measurement and the degree of *discrimination* at various points along the ability continuum. Also the extent to which the TCC is linear illustrates the degree to which the scale provides interval scale or linear measurement.

*Information* is the equivalent of variance explained, showing how effectively a measure captures the latent trait. *Information* can be calculated for each ability level. The greater the amount of *information*, the more precision with which the ability can be estimated.

IRT could improve tests used for diagnosing and monitoring people with dementia. By determining the *difficulty* of items within a scale it is possible to develop a hierarchy of item *difficulty* i.e. a list of questions from those with lowest *difficulty* (where the expected probability of a correct answer of 50% is reached at a low overall score) to those with highest *difficulty* (where the expected probability of a correct response of 50% is reached at a high score). This confirms the sequence of cognitive decline. Establishing a hierarchy of *difficulty* confirming the sequence of decline will allow clinicians and researchers to identify any deviations in the rate or sequence of cognitive decline from the usual trajectory of loss. Hierarchies of item *difficulty* may differ according to diagnosis or by country/region or by different translations of measures. Identifying unique sequences of cognitive decline for different forms of dementia could aid in diagnoses. Additionally being aware of the ordering of *difficulty* makes it possible for clinicians to tailor their assessments according to severity level, e.g., selecting less *difficult* items for patients with established dementia and the more *difficult* items for healthy elderly or those with mild or early stages of cognitive impairment [8].

IRT can also examine the sensitivities of the items within a measure. By examining the slope of the ICC the items *discrimination* can the assessed. The range of cognitive impairment at which the slope is the steepest is where that item will be maximally *discriminative*, differentiating well between various gradations of impairment and providing increased sensitivity to change. Determining the *discrimination* of items can reveal which items are most likely to expose changes in cognition and those with weaker *discriminatory* power that are unresponsive to such changes [9,10]. Looking at the item curves in relation to each other provides useful information on the breadth of measurement of an instrument. IRT can also identify key items which provide valuable information or whether any items within the scale are redundant, i.e. items with similar ICCs.

Applying IRT techniques to measures of cognitive functioning in dementia could have far reaching implications for clinicians and researchers leading to advancements in screening assessments and diagnosis, the charting of disease course and the measurement of change with disease progression and in response to treatment. In addition, IRT methodology will be useful to industry in the design of psychometric tests. IRT has been used to analyse clinical measures in several different fields: schizophrenia [11], depression [12], attachment [13], social inhibition [14] and quality of life [15]. IRT has also been used to examine ADL and Instrumental Activities of Daily Living (IADL) scales [16,17]. IRT methods have been successful in improving functional scales by establishing interval level measurement [18]; hierarchies of item *difficulty*[16,19,20]; *discrimination* of items [16,21]; as well as identifying ways of increasing measurement precision [18]. IRT analyses of measures of cognitive functioning in the general population have been described [22,23], including several papers with samples including some participants with dementia [24-28]. However, despite the strong theoretical basis outlined above for using IRT in people with dementia, there is limited published data. Therefore we performed a systematic review of the published studies that use IRT to revise or develop instruments assessing cognitive ability in people with dementia.

## Methods

### Search strategy

Published studies were identified through searches of Medline (including work in progress from 1946 until 5^th^ September 2013), Embase (1980 until 5^th^ September 2013), PsychInfo (1806 until 5th September 2013) and CINAHL (1981 until 5^th^ September 2013). Search filters included were keyword, title and abstract information. Search terms relating to IRT and dementia were combined. Articles with any combination of any of the IRT terms and any dementia term were reviewed. For full search strategy see Appendix 1. References of included studies were hand-searched and a forward citation search was performed on all included studies to establish all articles which cited them.

### Data extraction

A total of 384 articles were identified from this search. After duplicates were removed the titles and abstracts of 203 articles were screened by two independent researchers. 160 articles were excluded on review of title and/or abstract (for example, non IRT methods, IRT analyses of functional or other non-cognitive assessments). 43 articles considered to be relevant were retrieved and assessed for agreement with the following inclusion and exclusion criteria. Data were extracted from original studies onto forms which were refined following piloting.

**Figure 1:**
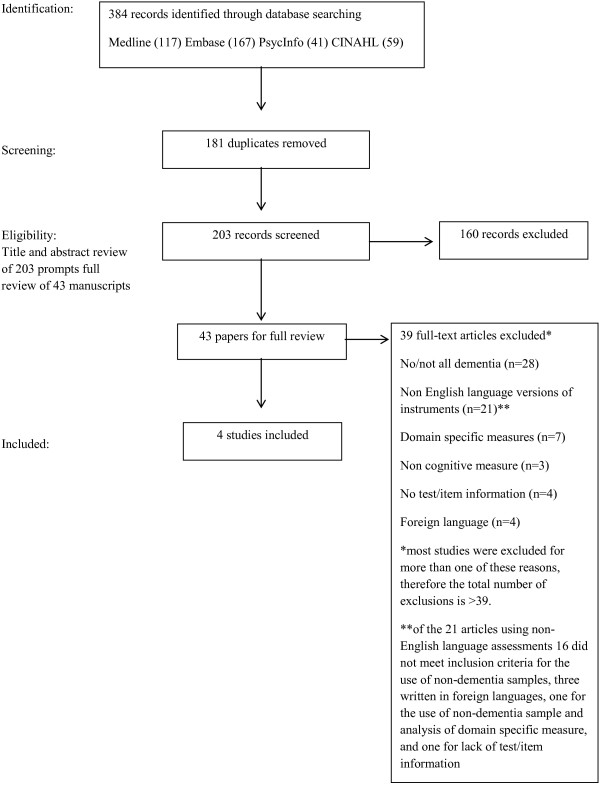
Flow diagram for manuscript selection.

Figure 1 shows the flow chart for this review.

### Inclusion criteria

This review aimed to include all published studies that applied item response theory methods to instruments with face validity for measuring global cognitive impairment in dementia. The initial search did not restrict results to those published in the English language.

### Exclusion criteria

(i) Unpublished studies, dissertations, theses, journal conference abstracts and poster presentations; (ii) studies using proxy reports as there is evidence of discrepancy between self-report and informant measures of cognitive functioning [29]; (iii) studies with participants without diagnosed dementia; (iv) studies without details of dementia diagnosis criteria or percentages of participants with dementia; (v) studies reporting IRT applications to domain specific measures of cognition rather than global cognitive functioning, for example the Boston Naming Test [30] used to measure confrontational word retrieval; (vi) studies that did not provide information on item level performance or overall test performance; (vii) studies examining non-cognitive scales, although studies which reviewed a range of outcomes had the results from the cognitive scales included; (viii) no language restrictions were made in the search, but non-English language articles were not included in the final review as they used non-English scales; (ix) use of Guttman scaling procedures [31].

While studies have found increased sensitivity of domain specific neuropsychological tests to early impairment than test of global cognition [32] this review chose to restrict its focus to IRT analyses of global cognitive instruments to increase clinical relevance as these are the most commonly used for testing in routine practice.

The decision to exclude Guttman scaling was based on the considerable evidence stating the inferiority of these methods in comparison to the more advanced item response methods [33]. The method was included in the search strategy; however, as some studies may have applied another method of analysis without indexing it and the exclusion of this term may have led to some relevant studies being overlooked.

Non-English language versions of cognitive measures were excluded. While several measures, most notably the MMSE [34], have been translated into many languages for use in different countries and cultures there are concerns over the cross-cultural validity. The language in which a test is administered can affect performance leading to a potential overestimation of cognitive impairment in individuals who do not speak English [35-37]. Differential Item Functioning (DIF) [38] can be applied to examine the effect of language bias of items and tests administered in different languages. For example, if patients of equal cognitive ability tested in English and Spanish have unequal probabilities of responding correctly to a particular item on a cognitive assessment, then the item functions differently with respect to language. The effect of different test languages of cognitive assessments has been examined in this way [27,39-43]. However these studies did not examine DIF in dementia populations and were therefore not included. Also the non-English language versions administered makes comparison with scales in English problematic because the semantic range of items cannot be assumed in translation [44], for example, repeating “No ifs, ands, or buts” corresponds to repeating “We put ones’ efforts all together and pull the rope” in the Japanese version of the MMSE [26] and to a tongue-twisting phrase “en un trigal habia tres tigres” (“there were three tigers in a wheat field”) in the Spanish version [45]. To avoid any potential confounding these articles were not included for full review [43]. The decision to exclude articles using non-English language assessments has no implications for the validity of cognitive testing in other languages.

## Results

Four cross-sectional studies met inclusion criteria, including 2,920 patients from six centers in two countries: Table 1 describes the characteristics of the studies reviewed. In total dementia aetiologies comprise 74.1% (2165) probable Alzheimer’s disease (AD), 9.3% (273) possible AD, 2% (60) Vascular dementia, 11.1% (325) mixed and other dementia. For individual studies see Table 1. Most patients fall within the moderate range of severity of dementia.

**Table 1:** Articles meeting inclusion criteria applying IRT methods to cognitive measures of dementia

**Study**	**Ashford *et al*.**[46]	**Mungas & Reed**[1]	**Gibbons *et al*.**[48]		**Benge *et al.*** [49]
**Country**	USA	USA	USA and UK		USA
**Setting**	Geriatric psychiatry	Two clinical sites of Alzheimer’s disease centre	Two community based samples from USA and UK		Alzheimer’s disease and memory disorders clinic
	Outpatient clinic				
**N**	86	1207	540 (US: 401, UK: 139)		1087
**Sex**	73.2% female	64.7% female	(US) 64% female		66.6% female
			(UK)75% female		
**Age**			(US)	(UK)	
**Mean**	74	76	82	84.7	75
**SD**	8	8.9	4.7	5.3	8.1
**Range**	53-91	39-100	> 75	>75	Not reported
**Etiology; *n *** **(%)**	Probable AD: 52 (60)	Probable AD: 592 (49.0)	UK: AD: 139 (100)		AD: 1044 (96)
	Possible AD: 34 (40)	Possible AD: 176 (14.6)	US: Probable AD: 338 (84.2)		MCI: 43 (4)
		Vascular: 60 (5.0)	Possible AD: 63 (15.7)		
		Mixed and other dementia: 325 (26.9)			
		No cognitive impairment: 27 (2.2)			
		Diagnosis deferred: 27 (2.2)			
**Dementia severity**	Mean MMSE=18	Mean MMSE= 17.7	US: Mean MMSE=19.6		Mean ADAS cog=31.2
	SD=7.1	SD=7.3	SD=4.9		SD=16.5
	Range=1-29	Range=0-30	Range=1-29		Range=not reported
		Mean BIMCT= 16.9	UK: Mean MMSE=16.5		
		SD=8.3	SD=5.5		
		Range=0-33	Range=0-25		
**Cognitive measure**	MMSE	MMSE, BIMCT	MMSE		ADAS-cog
**IRT method**	Item characteristic curve analysis	Two-parameter model	Item characteristic curve analysis		Samejima’s graded model
**Outcome**	Hierarchy of item difficulty and discrimination	Hierarchy of item difficulty of Global function scale. Investigation of linearity of MMSE, BIMCT and global function.	Hierarchy of item difficulty from 2 samples		Discrimination and information statistics on ADAS-cog test as whole, plus domains and subscales

AD = Alzheimer’s disease, MCI = mild cognitive impairment, MMSE = Mini Mental State Examination, ADAS-cog = Alzheimer’s disease Assessment Scale-Cognitive subscale, BIMCT = Blessed Information Memory Concentration Test.

Three cognitive tests (MMSE, ADAS-cog, BIMCT) and three different IRT methods (Item Characteristic Curve analysis, Samejima’s graded model, Two-parameter model) were used.

Ashford *et al*. [46] applied IRT techniques to identify the degree of AD severity at which individual items of the MMSE are lost and the rate at which they are lost at that level of severity. MMSE scores from 86 AD patients were analysed. Most people had moderately severe AD (mean MMSE score = 18).

A hierarchy of item *difficulty* was formed (see Table 2). Most *difficult* items were the three memory items and “Orientation to date” (which also tests recent memory), and “Serial sevens”. These findings suggest that the mental functions assumed to underlie performance of these items- memory and attention and calculation- are lost earliest in the progression of AD. Least *difficult* items, i.e. late loss, were “Verbal directions”, “Name pencil” and “Repeat nouns”. This pattern is consistent with the typical clinical course of AD starting with memory problems ultimately leading to problems with over-learned associations and early-learned verbal mimicking.

**Table 2:** **Item *difficulty *** **comparison across studies**

	**Ashford *et al*.**[46]**(MMSE)**	**Gibbons *et al*.**[48]**UK (MMSE)**	**Gibbons *et al.*** [48]**US (MMSE)**	**Mungas and Reed**[1]**(BIMCT/MMSE)**
**Truncated above upper limit**	Recall: tree	No ifs ands or buts		
	Recall: flag	Recall nouns		
**1st quartile (most difficult)**	Serial sevens: subtraction 5	Orientation to date	Orientation to date	Recall ‘42’ (BIMCT)
	Serial sevens: subtraction 3	Verbal directions	No ifs ands or buts	Recall ‘Market Street’ (BIMCT)
	Orientation to date	Intersecting pentagons	Intersecting pentagons	Recall ‘John’ (BIMCT)
	Recall: Ball	Serial sevens	Serial sevens	Recall ‘Chicago’ (BIMCT)
				Recall ‘Brown’ (BIMCT)
**2nd quartile**	Serial sevens: subtraction 4	Orientation to year	Recall nouns	Orientation to year (BIMCT/MMSE)
	Serial sevens: subtraction 2	Orientation to county/streets	Orientation to day	Orientation to month (BIMCT/MMSE)
	Orientation to day	Orientation to day	Orientation to year	Age (BIMCT)
	Orientation to county	Orientation to month	Orientation to season	
	Orientation to month		Orientation to month	
	Serial sevens: subtraction 1		Orientation to county/streets	
	Orientation to year			
	Orientation to season			
	Orientation to place			
	Orientation to floor			
**3rd quartile**	Orientation to city	Orientation to state/county	Orientation to address	Orientation to state (MMSE)
	Intersecting pentagons	Write sentence	Verbal directions	Type of work (BIMCT)
	Orientation to state	Orientation to	Write sentence	Count forward (BIMCT)
	Write sentence	Season	Orientation to place	Name watch (MMSE)
	No ifs ands or buts	Orientation to Address	Orientation to city	
	Name watch			
	Verbal directions: paper-on floor			
**4th quartile (least difficult)**	Close eyes	Repeat nouns	Orientation to state	Place of birth (BIMCT)
	Repeat: flag	Orientation to city/town/village	Close eyes	Name pencil (MMSE)
	Name pencil	Orientation to room	Name objects	Name (BIMCT)
	Repeat: ball			
	Repeat: tree			
	Verbal directions: paper-take in right hand			
	Verbal directions: paper-fold in half			
**Truncated below 0**		Close eyes	Repeat nouns	
		Name objects		

MMSE = Mini Mental State Examination, BIMCT = Blessed Information Memory Concentration Test.

Ashford *et al*. [46] and Gibbons *et al.*[48] test items divided into quartiles based on range of scores. Mungas and Reed [1] items divided into quartiles based on *difficulty* parameters.

Most *difficult* items were truncated above upper limit as *difficulty* estimates were above the upper limit. Easiest items were truncated below 0 as even this low level of ability most participants were able to answer correctly.

Some differences between MMSE versions between studies led to some discrepancies between items, e.g.: state/county.

For one of the least *difficult* items “Name pencil” participants with a score of 6.6 had a 50% probability of getting this item correct. At a score of 10 participants had an almost 100% chance of correctly identifying the pencil. This is in sharp contrast to the most *difficult* items “Recall nouns”. A participant with a score of 20 had approximately 25% chance of getting “Recall: Tree” correct. These recall items were answered incorrectly by approximately 83% of the participants.

Item *discrimination* was used as an index of the rate of loss. The most *discriminatory* items were: “Name pencil”, “Write sentence”, “Orientation to month”, “Name watch”, “Orientation to date”, “Orientation to year”, “Close eyes”. For these items there is a sharp cut-off of ability level at which the item was passed or failed. The items with the lowest *discriminative* power are those items lost earliest; “Recall: Tree” and “Recall: Flag”, and latest in disease course; “Verbal directions”. Due to these items assessing abilities which are either lost almost immediately or not until very late stages the rate of loss is not meaningful but the items do serve a useful purpose as they measure ability at either extreme of the MMSE scale.

Some limitations of this study include the fact that participants with possible AD were not excluded for sensitivity analysis. Also there was no explicit investigation of unidimensionality of the MMSE. However the item-by item analysis of the variability in AD implies that there is a strong unidimensional component in the course of AD. There was no report of who administered the MMSE to the participants and whether they were blind to diagnoses. This introduces potential for bias.

Mungas and Reed [1] analysed MMSE and Blessed Information Memory Concentration Test; BIMCT [47] scores from 1207 participants. A very broad range of cognitive impairment across the full range of MMSE and BIMCT scores was represented. Here IRT methods were employed to evaluate existing measures and to develop a new global functioning measure by selecting items from the existing scales with *difficulty* ranges spanning the breadth of ability levels to increase *discrimination* at all ability levels.

Items were recoded as dichotomized variables for analysis. Ordinal scale items such as “World backwards” in the MMSE were converted to a number of dichotomous items equal to the maximum score on this item, leading to total scores of 30 for the MMSE, 33 for the BIMCT. Cognitive tests were administered by a neuropsychologist, neuropsychology trainee or a trained psychometrist. The authors did not mention if these individuals were blind to diagnoses.

Test characteristic curves (TCCs) for both scales were generated. TCCs of the MMSE and BIMCT were distinctly non-linear, showing decreased *discrimination* at both ends of the ability continuum with linear measurement for moderate levels of impairment. This indicates relative insensitivity to changes in ability at each end of the ability spectrum.

A more linear brief composite instrument; ‘Global Function’ was created. Items were selected from the MMSE, BIMCT and a functional measure; Blessed-Roth Dementia Rating Scale (BRDRS). Items fitting uniform distribution of *difficulty* across the spectrum of ability measured by the three measures were selected. The new scale showed improved *discrimination* at low ability levels but due to the relative absence of high *difficulty* items in the MMSE, BIMCT and BRDRS the scale showed decreased *discrimination* at high ability levels. This illustrates the need to develop and add more *difficult* items to existing and new measures to decrease ceiling effects. The hierarchy of item *difficulty* of the cognitive items from this measure is provided in Table 2. While this measure included functional items which is beyond the scope of this review the most *difficult* items were memory items which is in line with previous findings.

Again there was no assessment of whether the items in the tests are sufficiently unidimensional for the use of IRT. It was not reported whether those who tested the participants were involved in the analysis.

Gibbons *et al*. [48] used IRT to compare the relative *difficulties* of MMSE items between people with AD living in the US and UK. The 401 US participants were comparatively less impaired (mean MMSE 19.6) than the 139 UK participants (mean MMSE 16.5).

There were some differences between items used for the two samples. Orientation to state and county items in US sample were substituted for orientation to county and 2 streets nearby for the UK cohort and the nouns to repeat and remember were also different for the two cohorts. Although these differences limit the direct comparison of difficulty between these items as the differences are limited to these items they are unlikely to explain the entire difference observed between the two samples. Reports indicate the interview structures did not differ between samples in any substantial way. For analysis all items which could have a score greater than one were dichotomized. All three nouns must be repeated and all stages of following the verbal directions must be carried out for these items to be scored as correct. “Recall nouns” was scored correctly if any one of the three nouns were recalled. Two points for “Serial sevens” were sufficient to be scored as correct. Therefore ability level was represented by the score of the 19 dichotomized items, excluding the score of the item under assessment resulting in score ranges from 0–18.

Gibbons *et al*. [48] established the relative *difficulties* of items for both cohorts, adjusted to an education level of high school or less.

### UK results

The most *difficult* items were “No ifs, ands or buts” and “Recall nouns”. At the uppermost score of 18 only an estimated 29% of participants could repeat the phrase “No ifs, ands or buts”.

The easiest items were “Close eyes” and “Name objects”. Here at an estimate of less than zero most participants could still answer correctly so again these estimates were truncated at 0. This reflects the relative simplicity of these items.

### US results

The most *difficult* items were “Orientation to date” and “No ifs, ands or buts”. At ability scores of 17.5 and 15.3 half of the participants could correctly identify the date and repeat “No ifs, ands or buts” respectively.

The easiest item was “Repeat nouns”. The ability score was again truncated at 0 indicating that even at this low level of ability most participants were able to answer correctly. “Name objects” and “Close eyes” were also relatively easy items.

Hierarchies of item *difficulty* for both UK and US samples are presented in Table 2. Five items; “No ifs, ands or buts” “Recall nouns”, “Orientation to state/county”, “Repeat nouns” and “Verbal directions” were significantly more *difficult* for the UK sample. While some items were more *difficult* for the US cohort the differences were not significant. A score of 15.6 was necessary for a UK participant to have a 50% chance of correctly responding to “Verbal directions” in comparison to a US participant having the same probability at a score of seven.

Additional analyses excluding ‘possible’ AD, MMSE items which differed between samples, and accounting for international differences in educational standards did not affect the results.

Attempting to control for the differing levels of severity between the samples, dementia severity (as assessed by the Dementia Rating Scale; DRS) along with age, education and gender were assessed as possible confounders of the relative *difficulty* of items. The relative *difficulty* of the items was not affected by the DRS. It is possible however that controlling for the DRS may not have been enough to compensate for the differences between the two groups.

The methodology applied here was rather robust given the additional analyses performed. However the researchers did not explicitly investigate unidimensionality of the instruments. The MMSE was administered at home by trained research interviewers for both cohorts. The scores used were taken from interviews preceding diagnosis which eliminated risk of bias. The diagnoses were not made by the researchers doing the analysis again limiting any potential bias.

Benge *et al*. [49] used IRT analyses to examine the measurement properties of the ADAS-cog across the spectrum of cognitive decline in AD. To determine the relationship between the level of impairment and the probability of achieving observed scores on the test as a whole and the test’s subscales scores from 1087 AD participants were analysed. 43 patients with mild cognitive impairment (MCI), diagnosed using Petersen *et al*., [50] criteria, were included. This is the only study to include MCI participants and although they account for only 4% of the sample it is worth keeping this difference in mind when interpreting the results. The mean ADAS-cog score was 31.2 indicative of moderate to severe dementia.

Benge *et al*. [49] assessed the unidimensionality of the ADAS-cog. Results from an exploratory factor analysis and confirmatory factor analysis confirmed the ADAS-cog as a one-factor scale.

The measure’s subscales were grouped into three domains: memory, praxis and language for analysis. Curves permitting the comparison of the domain performance across the spectrum of cognitive decline were created. These curves indicate that memory has most *discriminative* power at the relatively milder stages of decline in comparison to language and praxis which were maximally *discriminative* at the same stages later in the disease course.

Analysis of the 11 subscales showed “Word recall” to be the most *discriminative* at mild stages of disease making it the best indicator of mild cognitive decline. “Recall of instructions” remained relatively unaffected until the later stages of disease. Praxis and language subscale curves indicate that as with the domains, these subscales maximally *discriminate* at moderate levels of decline. The curves for “Ideational praxis”, “Construction” and “Word finding”, “Speech comprehension”, “Commands”, “Speech content” and “Naming” overlap considerably implying that they yield more or less the same information about patient’s stage of cognitive decline. All items *discriminate* well at moderate levels of severity.

*Information* analysis found perhaps not surprisingly the highest level of *information* is found at moderate levels of cognitive dysfunction. At this level a unit change in cognitive dysfunction represents a greater change in performance than the same change at either ends of the range. This indicates that the ADAS-cog as a whole has relatively high levels of *discrimination* and can differentiate between various degrees of ability at this moderate stage.

This study was the only one to report an assessment of unidimensionality prior to IRT analyses. This is an important assumptions underlying IRT theory and it is therefore important to have established that the ADAS-cog meets this assumption.

Analyses were carried out using the most recent of the patients’ ADAS-cog scores. It was not reported whether the researchers who carried out the analysis also scored and diagnosed the patients. This introduces some possibility of bias.

## Discussion

This is the first systematic review of studies applying IRT methods to the assessment of cognitive decline in dementia. This review employed a comprehensive search strategy and included a detailed narrative review of the studies meeting the inclusion criteria.

This review appraised four published studies of IRT analyses of the cognitive decline of 2,920 participants with dementia. The four studies reviewed provided demonstrations of the applicability of IRT to assessment of cognitive functioning in dementia.

### Item difficulty

Three of the four studies established a hierarchy of item *difficulty*[1,46,48]*.* Two of these hierarchies were of the MMSE items [46,48] and the third was of the Mungas and Reed ‘Global Function’ scale [1]. The dichotomization of MMSE items in Gibbons [48] decreased the ease at which direct comparisons of item *difficulties* between different studies could be made. In an attempt to equate the different range of MMSE scores across the studies items were divided into quartiles based on score ranges and *difficulty* parameters.

Table 2 shows that “Orientation to date”, “Recall nouns” and “Serial sevens” are consistently the most *difficult* items across studies. A clinician identifying problems with these tasks could expect the patient to develop further cognitive *difficulties* in the progression suggested by the hierarchies in Table 2. Generally the least *difficult* items were; “Name objects”, “Repeat nouns” and “Close eyes”. Problems with these items can help identify severe dementia. From a clinical perspective this information is very useful. It provides a clearer insight into decline than the traditional scoring method. *Difficult* items are very informative as it is likely that a patient with no *difficulties* here will not have limitations with other less *difficult* items. The items most consistently found the least *difficult* could be used in a similar fashion. It is likely that a patient unable to correctly respond to these items would have problems with most of the other items in the scale. In this way IRT analyses can identify key items from a scale that can quickly inform clinicians of a patient’s level of functioning, for example, a clinician could select from the most *difficult* items such as “Recall nouns” to identify potential early cognitive difficulties in the healthy elderly.

None of the studies attempted to determine whether the hierarchies of *difficulty* held at the individual level (ordering items in terms of *difficulty* does not necessarily mean the ordering is the same for every person; those with higher levels of ability may find one item more *difficult* than the other yet the ordering may be reversed for those with lower ability levels [46,51] by considering invariant item ordering (IIO). As invariantly ordered hierarchies are of great clinical value this should be included in future studies.

### Discrimination

Two studies determined item *discrimination*[46,49]. Table 3 summarises the findings from these papers, showing the most *discriminatory* items at the various stages of disease. High *discrimination* for low *difficulty* items indicates that the abilities assessed by these items are lost at an advanced stage and that these losses are rapid once this stage has been reached. For more *difficult* items high *discrimination* means that these abilities are lost in the early stages and quickly at this stage.

**Table 3:** **High *discrimination *** **items and disease stages**

	**Early disease/high difficulty**	**Moderate stages**	**Late disease/low difficulty**
**High discrimination**	“Orientation to date” (MMSE)	ADAS-cog	“Name pencil” (MMSE)
	“Word recall” (ADAS-cog)	“Ideational praxis” (ADAS-cog)	“Close eyes” (MMSE)
		“Construction” (ADAS-cog)	“Name watch” (MMSE)
		“Word finding” (ADAS-cog)	
		“Speech comprehension”	
		(ADAS-cog)	
		“Commands” (ADAS-cog)	
		“Speech content” (ADAS-cog)	
		“Naming” (ADAS-cog)	

Items with low *discrimination*; “Repeat nouns”, “No ifs, ands or buts”, “Orientation to day and season”, “Orientation to country, floor and city”, “Copy pentagons” also reveal valuable insights. For these items the range of scores in which participants respond either correctly or incorrectly is wider than high *discriminating* items. Either the abilities being measured by these items are lost with more variability or more gradually or the functions measured here are assessed less concisely by these items.

Including more items like “Word recall” and “Orientation to date” may help to detect changes in milder stages of the disease as these abilities are lost quickly at an early stage. For severe dementia the inclusion of simple repetition tasks or non-cognitive functioning tasks could help to introduce greater *discrimination* in this stage. Items such as recalling or recognizing one’s name, from the Severe Cognitive Impairment Rating Scale, measuring the ability of overlearned autobiographic memory, could be applied to broaden the range of assessment in cognitive instruments.

From a large battery of items those demonstrating the best *discrimination* across the disease course could be used to create an instrument to accurately measure patients in early and late stages. More precise assessment would lead to enhanced measurement of the rate of decline and improve predication of impending deterioration.

While these studies demonstrate the use of IRT to examine item *difficulty* and *discrimination* the investigation of item differences has also been addressed using classical test theory (CTT). Chapman and Chapman [52] identified the need to study these item parameters in their analyses of specific and differential deficits in psychopathology research, for example, specific deficits in schizophrenia or the analysis of domains or abilities which remain relatively intact in dementia. Chapman and Chapman’s analyses of differential deficits is rooted in classical test theory (CCT) and IRT, as a newer statistical model, offers alternative means of exploring the differential deficit problem. When examining differential deficits between different groups IRT, unlike CCT, can offer estimates of measurement error for different levels of cognitive ability, without having to conduct separate studies, and can establish whether different items or measures are equally *difficult*.

### Linearity and the assessment of change in severity

Two studies investigated whether the magnitude of cognitive dysfunction represented by each item on the cognitive scale was equal across the scale [1,49]. In a recent paper Balsis *et al*. [53] also drew attention to the limitations associated with the traditional method of measuring cognitive dysfunction with the ADAS-cog. This study was not included in the review as it did not provide information on the individual items or subscales however its analysis of IRT scoring of the ADAS-cog is worth noting. Balsis *et al.*[53] found that individuals with the same total score can have different degrees of cognitive impairment and conversely those with different total scores can have the same amount of cognitive impairment. These findings are supported by a similar study also failing to meet inclusion criteria due to some use of non-English language measures and a lack of information on test/item information [2]. Results indicate that participants with equal ADAS-cog scores had distinctly different levels of cognitive impairment. Equally, participants with the same estimated level of impairment had wide ranging ADAS-cog scores. The same differences in scores did not reflect the same differences in level of cognitive impairment along the continuum of test score range. Without equal intervals between adjacent test items change scores may reflect different amounts of change for subjects with differing levels of severity, or may fail to identify change at all [54]. Wouters *et al.*[2] revised the ADAS-cog scoring based on the results of this IRT analysis by weighting the items in accordance with their measurement precision and by collapsing their categories until each category was hierarchically ordered, ensuring the number of errors increase with a decline along the continuum of cognitive ability. Examining *difficulty* hierarchies of the error categories within the items revealed some disordered item categories. As the categories are only useful if they have a meaningful hierarchy of *difficulty* these disordered categories were collapsed until all categories were correctly ordered in hierarchies of *difficulty*. This revision resulted in a valid one to one correspondence between the summed ADAS-cog scores and estimated levels of impairment.

These studies demonstrate the potential to misinterpret test scores due to a lack of measurement precision. This is illustrated by Mungas and Reed’s examination of linearity of the MMSE, BIMCT and the ‘Global Function’ scale [1]. The findings of non-linearity of the MMSE and BIMCT indicate that a change in total score is less for a given specified change in ability at the two ends of ability distribution than it is in the middle of the ability distribution. For example, a two standard deviation change in ability from 3.0 to 1.0 reflects an approximate five point MMSE score loss, whereas the same degree of change from 1.0 to −1.0 represents a 15 point MMSE score loss. A similar pattern was found for the BIMCT. IRT methods can be used to create a scale with greater linearity by establishing item *difficulties*, as illustrated by the ‘Global Function’ scale [1]. The ‘Global Function’ scale shows promise of linear measurement throughout the majority of the continuum of ability. This new measure, along with any new IRT measure, would need to be cross-validated and directly compared to existing clinical instruments to ensure this test development technique is truly beneficial. It is worth noting that this measure also incorporates items assessing independent functioning. The inclusion of tasks such as these with meaningful variability even in the late stages of dementia could afford the test more *discriminatory* power increasing the information at this stage. While this review did not aim to include functional scales this study suggests that scales that combine cognitive and functional items, or concomitant use of both types, may provide added value. A limitation of this and many other cognitive functioning scales is the lack of items sensitive to very mild early stage of dementia. The inclusion of items capable of *discriminating* mild dementia could improve measurement properties in much the same way.

The measurement properties of a scale can impact the interpretation of clinical trials as change scores are used to determine the efficacy of interventions and treatments. A Cochrane review of AD pharmaceutical trials methods included ADAS-cog change scores to help ascertain the effectiveness of cholinesterase inhibitors [55]. Benge *et al.*[49] confirmed that the degree of cognitive ability symbolized by each point on the ADAS-cog was not uniform across the scale. A three point change in raw scores can represent a change in cognitive abilities ranging from 0.85 standard deviations of cognitive functioning (representing a change from a score of 4 to 1) to 0.14 standard deviations of cognitive functioning (from a score of 37 to 34).

The observation of differences between and within people may be greatly aided using an IRT approach. In clinical trials it is possible that these analyses will lead to an increased ability to correctly identify group treatment differences and to recognize responders and nonresponders to treatment.

### Information

Another advantage of IRT is the increased reliability it provides however, only Benge *et al.*[49] estimated the *information* parameter. The ADAS-cog has the highest level of *information* at moderate levels of cognitive impairment. At milder levels of impairment the *information* function remains low which indicates that the test domains; language, memory and praxis, and the measure as a whole do a relatively poor job *discriminating* among the different levels of impairment in the mild severity range. The same can be said about the severe levels of impairment. That moderate levels have the highest *information* function is unsurprising as the ADAS-cog was originally designed to measure moderate AD. Decreased *information* at mild and severe levels could affect the interpretation of the significance of the change scores at these levels of impairment.

This review excluded 28 studies using general populations, some of which included some dementia subgroups. In an effort to widen the scope of the review studies using general populations including some participants with dementia were looked at to determine if these dementia subgroups could be analysed separately. However it was determined that these papers failed to meet inclusion criteria for reasons beyond the sample characteristics, mostly for the use of non-English language measures, and therefore the authors of the papers were not contacted for further details. One such study analysed a Japanese version of the MMSE within a general population [26]. However the ordering of items was examined for the AD subgroup in isolation illustrating the sequence of cognitive decline. IRT analysis found the scale could be simplified with the removal of items showing similar ICCs and factor loadings, reflecting potential redundancy. “Naming” was deemed to be similar to “Three-step command” and was deleted along with “Read and follow instruction” showing similarity to “Repeat a sentence” and “Orientation to time” as its function was comparable to “Orientation to place”. The ordering from least to most *difficult* was “Three-step command”, “Registration”, “Repeat a sentence”, “Write a complete sentence”, “Copy drawings of two polygons”, “Delayed recall”, “Orientation to place” and “Serial sevens”.

21 studies were excluded for administering non-English measures. However, all except one were excluded for other reasons also (16 did not meet inclusion criteria for the use of non-dementia samples, three written in foreign languages, one for the use of non-dementia sample and analysis of domain specific measure, and one for lack of test/item information). The results of the single study [56] which was only excluded due to use of a Dutch version of the Baylor Profound Mental State Examination are discusses. Korner *et al*. [56] applied Mokken analysis and the one-parameter Rasch analysis in a validation study of the cognitive part of the Danish version Baylor Profound Mental State Examination. In doing so the relative *difficulty* of the test items were estimated. The *difficulties* of the 25 items were evenly distributed along the ability range with no redundant items. The least *difficult* items in this measure were; “What is your name?” and the repetition of the first word (one syllable). The most *difficult* item was the drawing of “Intersecting pentagons”. While the other studies administering such measures would not have been included for various other reasons there are data that may be informative [24,26,28,57].

While global cognitive instruments such as the MMSE are probably the most commonly used measure of cognitive functioning domain specific neuropsychological tests have been demonstrated to show increased sensitivity to early stages of cognitive impairment than measures of global cognition [32]. However of the seven studies applying IRT methods to domain specific measures identified [40,58-63] only one; Benge *et al*. [58] otherwise met inclusion criteria. This study’s findings were briefly discussed here. Temporal (“Day of month”, “Year”, “Month”, “Day of week” and “Season”), and spatial (“Name of hospital”, “Floor”, “Town”, “Country” and “State”) Orientation items of the MMSE, were analysed to determine their *difficulty* and *discrimination* parameters. The most *difficult* item was “Floor of hospital” and the least *difficult* item was “State”. The full order of item *difficulty* was; “Floor”, “Name of Hospital”, “Date”, “Day of Week”, “Year”, “Month”, “Season”, “Country”, “Town” and “State”. A relatively high level of ability (2.81SD) is required to have a 95% chance of correctly identifying the floor of the building which illustrates that knowing which floor of the hospital reflects a relatively high level of cognitive ability. Clinicians can use this sort of knowledge to help interpret the information they get from their assessments.

The spatial orientation items *discriminate* best at varying levels of cognitive ability with a wider range of *difficulties* assessed than the temporal items. Spatial items could be used to create a short scale sensitive to a relatively broad range of abilities. The temporal items assess a narrower breadth of abilities at a relatively modest degree of impairment and therefore would be best suited to identifying change within this range of cognition.

The value contributed by each item was examined to reveal key items and those whose function was largely redundant. “Year” and “Month” provide roughly the same information as they have similar levels of *discrimination* and *difficulty*, as do “State” and “Town”. Both item pairs provide no meaningful variability to the set of items. One item from each pair would be sufficient to capture the same information as both. “Date”, “Name of Hospital” and “State” together sample the range of cognitive abilities assessed by the orientation items and could together provide key information about a wide range of abilities.

### Some limitations of this review should be acknowledged

While the Preferred Reporting Items for Systematic Reviews and Meta-Analyses (PRISMA) guidelines were followed insofar as they were applicable for methodological studies the studies identified in this review did not allow a statistical summary or to perform a meta-analysis due to the variety of subjects, sites, diagnostic criteria and the varied statistical item response theory methods applied. The four studies cross a 20 year span with the earliest data collection and diagnoses in 1984 [46] with the most recent in 2002 [49]. This will affect criteria for diagnosing dementia. With mostly moderate ranges of dementia the studies also represented a rather restricted range of severity limiting the scope of the analysis as the findings cannot be extrapolated to mild or severe dementia.

IRT analyses assume unidimensionality which limits its application to measures assessing a single latent construct. However only one study reviewed here explicitly assessed unidimensionality prior to IRT analyses [49].

Three of the four studies failed to report who administered the test to participants and whether these individuals were blind to the diagnoses [1,46,49]. This introduces some potential bias in these studies.

This review was limited to analyses of only three global cognitive function; MMSE, BIMCT and ADAS-cog. This was a consequence of the articles meeting inclusion criteria. However, an analysis of the Baylor Profound Mental State Examination, while not reviewed due to use of a Dutch version, was mentioned in the discussion [56].

With the exception of Mungas and Reed [1] all studies solely included patients with Alzheimer’s disease. This could have an impact on findings as there should be a different pattern of decline between different aetiologies. Of the excluded articles one included patients with amyotrophic lateral sclerosis and behavioural variant frontotemporal dementia which would have expanded the scope of this review [64]. However this study failed to provide data on the measure of cognition in isolation from the other outcomes studied and for this reason was excluded.

## Conclusion

This systematic review of IRT use in cognitive tests in people with dementia found only four relevant published papers. These include heterogeneous populations, with widely varying sample sizes, different methods of dementia diagnosis (and inclusion of possible dementia or MCI), and samples are mostly derived from specialist clinical populations, with a risk of inclusion bias. Most participants had Alzheimer’s dementia of moderate severity, and were resident in the United States, so the relevance of this method to other subtypes of dementia, and other countries, cannot be determined. Different cognitive tests, and IRT methods, were used, and different statistics were reported. However, the studies show that IRT can demonstrate which items within scales are most *difficult*, and *discriminatory*, at different severities of dementia. IRT analyses can also be used to reveal non-uniform distances between scale scores and facilitate the creation of scales with enhanced measurement properties allowing more accurate assessment of change across the ability spectrum.

There is a need for more IRT analyses of cognitive scales used to assess dementia. These should include standard methodologies, and report item *difficulty* and *discriminatory* statistics along with a measure of *information* and an assessment of linearity of measurement. They should include large numbers, from a variety of countries (both English speaking and non-English-speaking), different dementia subtypes, the full range of severity of dementia, and a wider range of cognitive tests, focusing on those that are widely used in clinical practice. This will allow refinement of these tools to improve the information provided to clinicians on how performance on items within the scale is informative at different stages in dementia.

### Appendix 1

#### Search strategy

##### PsychoInfo

IRT terms:

1. Item response theory/or “difficulty level (test)”/or “item analysis (statistical)”/

2. Mokken.tw.

Dementia terms:

3. dementia/or dementia with lewy bodies/or vascular dementia/ or Alzheimer’s disease/

4. dementia.tw. or

5. semantic dementia/

##### Medline

IRT terms:

1. “item response theory”.tw. or

2. IRT.tw. or

3. “item response analysis”.tw. or

4. “modern testing theory”.tw. or

5. (cumulative adj2 structure).tw. or

6. “scale construction”.tw. or

7. “guttman scaling”.tw. or

8. “guttman scale”.tw. or

9. Mokken.tw. or

10. rasch.tw or

11. uni?dimensional*.tw. or

12. “cumulative order”.tw. or

13. “item characteristic curve”.tw.

Dementia terms:

14. dementia/or Alzheimer disease/or dementia, vascular/or frontotemporal lobal degeneration/or lewy body disease

15. dementia.tw.

##### Embase

IRT terms:

1. “item response theory”.mp. or

2. Mokken.mp. or

3. IRT.mp. or

4. “modern testing theory”.mp. or

5. (Cumulative adj2 structure).mp. or

6. “scale construction”.mp. or

7. “guttman scaling”.mp. or

8. “guttman scale”.mp. or

9. Rasch.mp. or

10. Uni?dimensional.mp. or

11. “cumulative order”.mp. or

12. “item characteristic curve”.mp. or

13. “item response analysis”.tw.

Dementia terms:

14. Dementia/or Alzheimer’s disease/ or frontotemporal dementia/or multiinfarct dementia/

15. Dementia.tw. or

16. Diffuse Lewy body disease/

##### CINAHL

IRT terms:

TX (“item response theory” or “item response analysis”) OR TX (Mokken or IRT) OR TX (“modern testing theory” or rasch) OR TX (“scale construction” or “item characteristic curve”) OR TX (“guttman scaling” or “guttman scale”) OR TX “cumulative order”

Dementia terms:

TX (Dementia or “Alzheimer’s disease”) OR TX (“vascular disease” or “frontotemporal dementia”) OR TX “lewy body disease”.

## Abbreviations

IRT: Item response theory; CINHAL: Cumulative index to nursing and allied health Literature; MMSE: Mini mental state examination; ADAS-cog: Alzheimer’s disease assessment scale-cognitive subscale; BIMCT: Blessed information memory concentration Test; ICC: Item characteristic curve; ADL: Activities of daily living; TCC: Test characteristic curve; IADL: Instrumental activities of daily living; AD: Alzheimer’s disease; BRDRS: Blessed-Roth dementia rating scale; DRS: Dementia rating scale; MCI: Mild cognitive impairment; IIO: Invariant item ordering; PRISMA: Preferred reporting items for systematic reviews and meta-analyses.

## Competing interests

The authors declare that they have no competing interests.

## Authors’ contributions

SM devised the search strategy, performed the literature review, drafted the manuscript, and conducted the data collection and analysis. EJA, SDS and JMS contributed to design, developed inclusion and exclusion criteria and contributed substantially to revisions of the paper for scientific content. JMD conducted literature review and selected studies for inclusion. All authors read and approved the final manuscript.

## Pre-publication history

The pre-publication history for this paper can be accessed here:

http://www.biomedcentral.com/1471-244X/14/47/prepub
